# Neonatal Circumcision for HIV Prevention: Cost, Culture, and Behavioral Considerations

**DOI:** 10.1371/journal.pmed.1000219

**Published:** 2010-01-19

**Authors:** Seth C. Kalichman

**Affiliations:** Department of Psychology, University of Connecticut, Storrs, Connecticut, United States of America

## Abstract

Seth Kalichman discusses a new study that finds neonatal circumcision in Rwanda to be a cost-saving HIV prevention strategy.

**Linked Research Article**

This Perspective discusses the following new study published in *PLoS Medicine*:

Binagwaho A, Pegurri E, Muita J, Bertozzi S (2010) Male Circumcision at Different Ages in Rwanda: A Cost-Effectiveness Study. PLoS Med 7(1): e1000211. doi:10.1371/journal.pmed.1000211

Decades of epidemiological studies and three carefully controlled randomized clinical trials have definitively shown that male circumcision (MC) reduces risks for HIV transmission from women to men by as much as 55% [1]. Male circumcision is therefore more protective against HIV transmission than even the most promising vaccines and topical microbicides. The protective biological mechanisms of MC are most likely a combination of removing HIV vulnerable cells that are present at high densities in the foreskin, particularly Langerhans cells, keratinization of mucous membranes, and reduction of penile trauma during intercourse. There is also evidence that MC offers protection against other sexually transmitted infections, further reducing the risk of HIV acquisition and transmission [2]. Although MC offers little, if any, direct protective benefits to women who engage in vaginal or anal intercourse with HIV infected men, or to male receptive anal intercourse partners of HIV-positive men, population-level reductions in HIV prevalence among men will ultimately lead to fewer infections in their sex partners.

Efforts to scale up MC for HIV prevention have thus far focused on promoting circumcision for young adult men, and there is ample evidence for high levels of acceptability in this group [3],[4]. Cost-effectiveness studies show that the monetary expenditures of scaling up MC in southern Africa are offset by dramatic savings in productivity and health care expenditures. For example, Kahn et al. [5] found that full-scale coverage of MC in South Africa's Gauteng province, which has an HIV prevalence of over 25%, would save $2.4 million over a 20-year period. Because MC is a partially effective HIV prevention strategy, its effects are cumulative over men's sexually active lifetimes and will, therefore, have most impact when implemented prior to sexual debut [6]. Neonatal circumcision is safer than circumcision in adulthood, carrying lower risks for surgical errors, infection, and other adverse events. As with adult MC, there is also evidence that neonatal MC has high acceptability for HIV prevention [7]. Circumcising male infants has therefore emerged as an important consideration in policy discussions for scaling up MC for HIV prevention.

## The Cost-Effectiveness of Neonatal Male Circumcision

In a study published in this issue of *PLoS Medicine*, Agnes Binagwaho and colleagues conducted a comparative cost-effectiveness analysis of neonatal, adolescent, and adult MC scale-up in Rwanda, a country with a moderate adult HIV prevalence of about 3% [8],[9]. The study used the perspective of the Rwandan government as the health care payer and used standard costs associated with the procedure as well as costs associated with HIV testing, treatment, and care. The model was based on current estimates of HIV incidence in Rwanda and an estimated 55% protective effect of MC. Analyses once again showed that MC is a cost-saving HIV prevention intervention, with both neonatal and adult MC saving Rwanda resources for each HIV infection averted. Furthermore, neonatal MC is less expensive than adult and adolescent MC, rendering greater dividends despite the time lag between the procedure and averted infections.

As with any HIV prevention strategy, the benefits of MC are most apparent when HIV incidence is highest. However, sensitivity analyses showed that neonatal MC remains cost saving even under very low estimates of HIV incidence. Binagwaho et al. conclude that providing universal access to MC, including neonatal MC, in conjunction with other effective HIV prevention interventions will reduce the overall cost of effectively fighting severe HIV epidemics driven by heterosexual transmission.

## Cultural Factors Can Undermine the Public Health Impact of MC

The case for MC, including neonatal MC, for HIV prevention is biologically and medically compelling. However, as with any other public health intervention, the effectiveness of MC will be determined by access and uptake. Cost-effectiveness analyses such as those reported by Binagwaho et al. illustrate the public health utility of increased access to neonatal MC. However, uptake may turn out to be a far greater challenge than can be estimated in cost-effectiveness analyses.

Rwanda was an interesting choice for a neonatal cost-effectiveness analysis because the country may represent a best case scenario for neonatal MC scale-up in Africa. More than 90% of the Rwandan population is Christian, and in Christianity there is little if any religious and cultural meaning attached to MC. Thus, while the country does not already routinely circumcise at birth, there will likely be little resistance to scaling up neonatal MC. Indeed, Rwanda has already initiated a national MC program that focuses on infants as one of its first priority populations [9].

Resistance to neonatal MC will surely be greater in African cultures where MC in young men is central to concepts of masculinity and maturity, often in places where HIV prevalence is much higher than Rwanda. In South Africa, for example, Xhosa communities circumcise young men in a rite of passage that is key to gender definitions, marking the transition from boyhood to manhood. Cultural beliefs and conceptualizations of masculinity, including what it means to be a man, are turned upside down when neonatal MC is introduced to cultures where MC is a pubertal rite of passage [10]. These cultural realities may at least in part account for why Rwanda is rapidly scaling up MC programs for HIV prevention whereas South Africa, a country with nearly four times the national HIV prevalence of Rwanda, remains stalled on implementing MC. The power of cultural and religious beliefs is readily apparent to orthodox Jews or Muslims who contemplate the ramifications of any public health recommendation that opposes MC. For example, when New York City's health department launched a public health campaign to oppose an ancient form of ritualistic neonatal circumcision, ultra-Orthodox Jewish leaders held a rally against the campaign [11]. Recognizing and understanding the cultural and religious beliefs attached to MC in areas most seriously affected by HIV/AIDS will be crucial in the successful scale-up of this effective HIV prevention strategy.

## Ignoring Behavioral Factors Can Undermine MC for HIV Prevention

Cultural and religious beliefs are not the only nonbiological factors to consider in scaling-up neonatal MC. Anticircumcision groups have long existed and are increasingly vocal as MC programs for HIV prevention are promoted [12]. Anticircumcision groups resemble other antiscience and antimedicine extremists including AIDS denialists who refute public health realities to maintain entrenched belief systems [13].

Another behavioral consideration in the scale-up of MC is the potential for risk compensation. In other words, men who elect MC to reduce their risks for HIV may subsequently stop using condoms and possibly increase their number of sex partners in response to their lower perceived risk [14]. While risk compensation following MC may occur, the evidence thus far is mixed [14]–[16]. It is possible that boys who grow up circumcised will not experience compensatory behavior because they will not undergo reductions in risk perception. However, an increase in beliefs that a man's circumcision status determines his vulnerability to HIV will likely shift social norms, with the potential for community-wide risk compensation. The contextualization and framing of MC must therefore be tailored to each individual culture to avoid adverse behavioral ramifications of implementing neonatal MC [17]. The slow uptake of MC may be due to a failure to take into account the cultural and behavioral issues surrounding MC. This slow pace risks offsetting the potential long term impact of MC for HIV prevention.

## Conclusion

MC offers one of the few available effective HIV prevention interventions. Scaling up MC in southern Africa has the potential to stem entire HIV epidemics, saving countless lives. Lifetime protection against HIV, and therefore reductions in population levels of HIV/AIDS, can be realized when circumcision occurs prior to sexual debut. The cost-savings of neonatal MC are compelling and suggest that implementation is economically feasible in developing countries hit hardest by HIV/AIDS. Neonatal MC should therefore be considered a priority in comprehensive HIV prevention plans for southern Africa.

## Abbreviations

MC: male circumcision
